# Early-life stress exposure and large-scale covariance brain networks in extremely preterm-born infants

**DOI:** 10.1038/s41398-022-02019-4

**Published:** 2022-06-18

**Authors:** Femke Lammertink, Martijn P. van den Heuvel, Erno J. Hermans, Jeroen Dudink, Maria L. Tataranno, Manon J. N. L. Benders, Christiaan H. Vinkers

**Affiliations:** 1 Department of Neonatology, University Medical Center Utrecht, Utrecht University, Utrecht, The Netherlands; 2 Department of Complex Trait Genetics, Center for Neurogenomics and Cognitive Research, Amsterdam Neuroscience, Vrije University Amsterdam, Amsterdam, The Netherlands; 3 Department of Child Psychiatry, Amsterdam Neuroscience, Amsterdam UMC, Amsterdam, The Netherlands; 4 Donders Institute for Brain, Cognition, and Behaviour, Radboud University, Nijmegen, The Netherlands; 5 Department of Cognitive Neuroscience, Radboud University Medical Center, Nijmegen, The Netherlands; 6 Department of Anatomy & Neurosciences, Amsterdam UMC (location Vrije University Amsterdam), Amsterdam, The Netherlands; 7 Department of Psychiatry, Amsterdam UMC (location Vrije University Amsterdam), Amsterdam, The Netherlands

**Keywords:** Neuroscience, Psychiatric disorders

## Abstract

The stressful extrauterine environment following premature birth likely has far-reaching and persistent adverse consequences. The effects of early “third-trimester” ex utero stress on large-scale brain networks’ covariance patterns may provide a potential avenue to understand how early-life stress following premature birth increases risk or resilience. We evaluated the impact of early-life stress exposure (e.g., quantification of invasive procedures) on maturational covariance networks (MCNs) between 30 and 40 weeks of gestational age in 180 extremely preterm-born infants (<28 weeks of gestation; 43.3% female). We constructed MCNs using covariance of gray matter volumes between key nodes of three large-scale brain networks: the default mode network (DMN), executive control network (ECN), and salience network (SN). Maturational coupling was quantified by summating the number of within- and between-network connections. Infants exposed to high stress showed significantly higher SN but lower DMN maturational coupling, accompanied by DMN-SN decoupling. Within the SN, the insula, amygdala, and subthalamic nucleus all showed higher maturational covariance at the nodal level. In contrast, within the DMN, the hippocampus, parahippocampal gyrus, and fusiform showed lower coupling following stress. The decoupling between DMN-SN was observed between the insula/anterior cingulate cortex and posterior parahippocampal gyrus. Early-life stress showed longitudinal network-specific maturational covariance patterns, leading to a reprioritization of developmental trajectories of the SN at the cost of the DMN. These alterations may enhance the ability to cope with adverse stimuli in the short term but simultaneously render preterm-born individuals at a higher risk for stress-related psychopathology later in life.

## Introduction

Preterm-born infants experience a persistently higher risk for anxiety and depressive disorders across the life span [1]. Much of the neural foundation of socio-emotional development is laid down in the fetal and neonatal period with profound morphological changes in brain regions involved in saliency, i.e., perceiving and responding to threat and stress [2, 3]. These changes during “third-trimester” development do not occur in isolation, and there is a high level of developmental coordination or synchronized maturation within and between large-scale brain networks [4]. Stress-provoking early-life experiences following preterm birth might have a programming effect on sensitive, still maturing, neuronal brain networks [2]. Hence, during this critical period of brain development, the third trimester in preterm infants could be viewed as a period of significant adversity which may lay the foundation for a lifelong increased risk for a wide range of psychiatric disorders.

Over recent decades, studies have indicated that brain development is delayed following preterm birth [5–10], but an increasing number of studies emphasized the degree of differential vulnerability with the advancement in brain development in a region-specific manner [10–13]. For example, patterns of accelerated functional development of brain regions may occur that are pivotal for detecting and responding to salient stimuli, including the amygdala and insula [14–16]. Similar to sensory regions, the development of which is regulated by activity-dependent modification in cellular events [17, 18], the brain’s salience processing networks appear to be regulated by stressful environmental input [19]. Thus, early environmental input may lead to a reprioritization of developmental trajectories [20]. Such a developmental trade-off may be adaptive in the immediate extrauterine environment, but, as the environment changes, it may become detrimental later in life [20, 21].

The coordinated growth of brain regions—maturational covariance—provides insight into the topographical organization of the developing brain and, according to previous studies [22, 23], reflects patterns of the functional organization of large-scale brain networks. The accelerated maturation of salience-related brain regions might be detrimental to other networks, providing a potential mechanism for malleability in functional outcome. Examining the developmental trajectory of gray matter covariance and the role of early-life stress exposure provides novel insight on macrostructural properties of large-scale brain networks and may, in turn, help to better understand the developmental origins of resilience and vulnerability following preterm birth.

Therefore, the current study investigates the impact of early-life stress exposure, as indicated by the number of invasive procedures, on ex utero “third-trimester” development of network-oriented covariance patterns in a population of extremely preterm infants (<28 weeks of gestation). During sensitive postnatal periods— specifically the “third-trimester”—the extrauterine brain development enables us to probe the effects of early-life stress on large-scale brain networks’ maturational covariance through structural MRI scans obtained at 30 and 40 weeks of gestation. Specifically, we focused on three canonical brain networks that are pivotal in the central response and regulation of stress: the default mode network (DMN; involved in self-referential and autobiographical memory functions), executive control network (ECN; involved in cognitively demanding tasks such as decision-making), and salience network (SN; important for detecting salient internal and environmental stimuli) [3]. Based on prior studies [21], we anticipated a higher maturational coupling of the salience system, including the amygdala, for infants exposed to more stress, which might come at the cost of other networks.

## Materials and methods

### Participants

The current study included a total of 180 extremely preterm-born infants. Specifically, preterm-born infants (gestational age <28 weeks) were all admitted to the Neonatal Intensive Care Unit (NICU) at the Wilhelmina Children’s Hospital, Utrecht, The Netherlands, and scanned between 28–32 and 39–42 post-menstrual age (cohort 2008–2019). The data collection was part of standard clinical care. Preterm infants with chromosomal and/or congenital anomalies, a potential confound in brain development studies, were excluded. Clinical information for all included infants is summarized in Table 1. Permission from the medical ethical review committee of the University Medical Center Utrecht (METC Utrecht) was obtained.

**Table 1:** Sample demographic and neonatal clinical details (*n* = 180).

	Total (*N* = 180)	Low stress (*n* = 90)	High stress (*n* = 90)	*P* value
Age at birth, mean ± SD, weeks	26.64 ± 0.99	26.86 ± 0.95	26.29 ± 0.99	*P* < 0.01
Age at scan, mean ± SD, weeks				
30 weeks	30.00 ± 0.97	30.57 ± 0.77	31.00 ± 0.96	*P* < 0.001
40 weeks	41.00 ± 0.90	41.00 ± 1.02	41.00 ± 0.59	ns
Sedation during scan (yes/no)				
30 weeks	127/53	66/24	61/29	ns
40 weeks	170/10	88/2	82/8	ns
Gender, female/male, *n*	78/102	42/48	36/54	ns
Birthweight *z*-score^a^, mean ± SD, g	−0.45 ± 1.40	−0.42 ± 1.44	−0.38 ± 1.37	ns
Invasive procedures^b^, median (range)	0.03	−0.52	0.68	*P* < 0.001
	(−3.28 − 2.51)	(−3.28–0.02)	(0.03–2.51)	
Days of morphine, mean ± SD	3.14 ± 5.80	2.10 ± 4.49	4.15 ± 6.72	*P* < 0.05
Prenatal corticosteroids (yes/no)	164/16	84/6	80/10	ns
Postnatal corticosteroids (yes/no)	54/126	21/69	33/57	*P* < 0.05
Intraventricular hemorrhaging (yes/no)	59/121	27/63	32/58	ns
Necrotizing enterocolitis, *n*	9	4	5	ns
Retinopathy of prematurity, *n*	60	18	42	*P* < 0.01
Meningitis, *n*	1	0	1	ns
1 min APGAR score, median (range)	5 (0–9)	6 (0–9)	5 (0–9)	
5 min APGAR score, median (range)	8 (0–10)	8 (0–10)	7 (2–9)	

^a^Dutch Perinatal registry reference data (Perined [103]).

^b^Centralized and standardized cumulative sum of invasive procedures during the stay in NICU Statistical significance was assessed with either a *T* test (for continuous data) or a Kruskal–Wallis test (for ordinal data).

### MRI acquisition and preprocessing

MRI data included the examination of 3T structural T_2_ images (3T Achieva MR scanner). Images were obtained during a 35-minute scanning session, using a Turbo Spin Echo (TSE) sequence, using parameters: TR = 6112 ms, TE = 120 ms, voxel resolution in millimeters 0.53 × 0.64 × 2 for 30 weeks and TR = 4851 ms, TE = 150 ms, voxel resolution in millimeters 0.78 × 0.89 × 1.2 for 40 weeks. Infants were immobilized by wrapping them into a vacuum cushion. MiniMuffs (Natus Europe, Münich, Germany) and earmuffs (EM’s kids Everton Park, Australia) were used to reduce noise and the infant’s propensity to move during image acquisition. Prior to scanning, preterm-born infants scanned at 30 weeks were either sedated with 30 mg/kg oral chloral hydrate or not sedated at all, whereas infants scanned at 40 weeks were all sedated with 50–60 mg/kg oral chloral hydrate. If the infant woke up, scanning was halted, and attempts were made to re-settle the infant without taking them out of the patient immobilization system. A neonatologist was present at all times during the examination.

Volumetric segmentation of MRI data was performed using the structural pipeline from the developmental human connectome project (dHCP; http://www.developingconnectome.org/). Briefly, structural scans were pre-processed by first running bias correction using the N4 algorithm [24]. These images were then brain extracted using BET 2 from FSL. Segmentation of the T2 images was performed using the DRAW-EM algorithm [25]. More specifically, manually labeled atlases, annotated by an expert neuroanatomist [26], were registered to the volume, and their labels were fused to the subject space to provide structure priors. Segmentation was then performed with an Expectation-Maximization scheme that combines the structure priors and an intensity model of the volume. The current study included 32 out of 87 labels.

### Early-life stress

Early-life stress was quantified according to prior published studies [27–29]. Specifically, the total number of invasive procedures was summated during the first weeks after birth up until the first scan at 30 weeks of gestation. The importance of the first weeks is emphasized because the majority of neonatal invasive procedures occur within the first weeks, with the greatest average daily exposure in the first 14 days after birth. In addition, some studies reported a more potent effect of early stress (i.e., first few weeks after birth) versus late stress (i.e., around term equivalent age) on brain development [30–32]. We, therefore, collected all clinical factors and treatments that were recorded throughout the first weeks of an infants’ NICU stay, such as the number of skin-breaking procedures, including heel lance, intravenous and central line insertion, intramuscular injection, and chest tube insertion, as well as days of cerebral monitoring and mechanical ventilation, and suctioning of mouth and nose. The cumulative sum of invasive procedures was weighted on the duration of NICU stay. See Supplementary Fig. S1 for the distribution of stressful procedures. The distribution of NICU-related invasive procedures observed in the current study are is in line with previously reported numbers (e.g., skin-breaking procedures [33, 34], days of mechanical ventilation [31, 35, 36]). For subsequent analyses, infants were grouped into low (lowest 50%) or high (highest 50%) stress exposure (for cut-off see Table 1). To ascertain the robustness of findings, we additionally (1) split the dataset on zero-mean so that infants with a stress score below zero were considered low stress, while infants with a stress score above zero were considered as high stress, and (2) looked at dose-dependent effects by dividing the sample into low (<33.3%), mild (33.3–66.6%) and high (>66.6%) stress exposure.

### Construction of maturational covariance network

Maturational covariance networks (MCNs) were constructed using previously published procedures (see for example [37, 38]). First, 32 volume measurements, 16 regions per hemisphere, were extracted from the segmented structural images. The regions of interest are all key regions of either the SN (i.e., amygdala, anterior cingulate cortex, insula, thalamus, subthalamic and lentiform nucleus [39, 40]), ECN (i.e., frontal lobe, parietal lobe, cerebellum, and caudate nucleus [41–43]), or DMN (i.e., posterior cingulate gyrus, hippocampus, anterior/posterior parahippocampal gyrus, and anterior/posterior fusiform [44, 45]). Second, a linear regression was used to remove the effects of covariates, including gender, total brain volume, gestational age, age at scan, degree of brain injury (i.e., intraventricular hemorrhage), surgeries, administration of pre- and postnatal corticosteroids (i.e.,, accelerates lung maturation), and days of morphine, using the package brainGraph in R (version 3.0.2 [46]). The subsequent studentized residuals were used for the construction of the MCNs. More specifically, the difference in volumetric values between *t*_*2*_ and *t*_*1*_ was computed by dividing the difference, normalized on *t*_*1*_ volumes, by the difference in weeks between the two-time points (i.e., 30 and 40 weeks of gestation). Then, the annualized difference scores were used to generate a 32 × 32 association matrix across subjects, with each entry r_*ij*_ defined as the inter-regional Pearson’s correlation coefficient between the studentized trajectory of gray matter volumes of region *i* and *j*. The diagonal cells were set to zero. Raw correlations were all transformed to *z*-scores by Fisher’s z-transformation. In line with prior studies [47], maturational coupling was defined as the similarity in the trajectory of gray matter development.

### Data analysis

#### Network-level comparison

The degree of maturational coupling was quantified within and between regions of three well-established networks of functionally and structurally connected (sub)cortical brain regions: SN, DMN, and ECN.

Nonparametric permutation testing was used to examine differences in maturational coupling between low and high-stress groups (median split, see Table 1). Subjects were shuffled 5000 times between each group, and each time a new association matrix was obtained between randomized stress labels and volumetric scores. The association matrices were thresholded (i.e., ensuring equal network density by selecting the strongest connections [48]) and binarized. Edges with a weight below the threshold are binarized to 1 and edges with a weight above the threshold are binarized to 0. Then, the total number of within- and between-network connections were computed. The differences in the number of network connections between randomized stress labels represented a permutation distribution of difference under the null hypothesis. The actual between‐group differences in network connections (Delta, hereafter *∆*) were tested against the obtained permutation distribution, and a two‐tailed *P* value was calculated based on its percentile position. As there is no gold standard for density threshold, we applied a range of thresholds (0.01 ≤ K ≤ 0.5, 0.01 increments) to each association matrix. Importantly, with increasing density levels, covariance patterns become more random (i.e., modularity, small-worldness) and are likely non-biological. Therefore, we limited the range of density levels to 0.10 ≤ K ≤ 0.30, 0.01 increments. We report findings at a single network density (20%) and show robustness by replicating findings across density levels. All comparisons were corrected for multiple testing using false discovery rate correction [*p*-FDR < 0.05] (R base package stats, version 4.1.0). Positive values indicate a higher coupling for high-stress-exposed infants relative to low-stress-exposed infants.

Secondary analyses were conducted to determine which regions drive network-level stress effects. We converted the maturational covariance networks to *Z*-scores using Fisher’s r-to-z-transformation (R package cocor, version 1.1–3 [49]). The MCN maps for the high- and low-stress-exposed group were then statistically compared at group-level using the *Z*-statistic (corrected for multiple testing using FDR [*p*-FDR < 0.05] (R base package stats, version 4.1.0)). Spurious correlations (*r* < 0.10) were removed prior to transformation.

## Results

### The effects of early-life stress on network-level maturational covariance

**Fig. 1:**
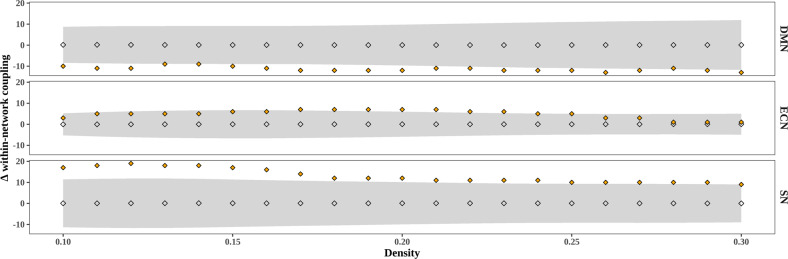
Difference in within-network maturational coupling between preterm-born infants exposed to high versus low stress (median split) across density levels. Positive values show high stress >low stress and negative values show low stress >high stress. Gray diamonds depict the permutation distribution of average maturational coupling, gray bands depict 95% confidence interval, and orange diamonds depict the group-level difference score.

Stress exposure during the first weeks of life resulted in a significant lower within-network maturational coupling (i.e., their difference in network organization as measured from 30 to 40 weeks of gestation) of the DMN in high-stress-exposed infants (*n* = 90) compared to low-stress-exposed infants (*n* = 90) (∆ = −12, *p-FDR* < 0.01, see Fig. 1). These findings suggest that stress may lower similarity in the rate of volumetric development between DMN regions. In contrast, stress resulted in the higher within-network maturational coupling of regions of the SN (∆ = 12, *p-FDR* < 0.01) and ECN (∆ = 7, *p-FDR* < 0.05), suggesting that stress may accelerate the maturational coupling of brain regions of these other higher-order networks. Notably, the ECN effects were not consistently replicated across density levels.

**Fig. 2:**
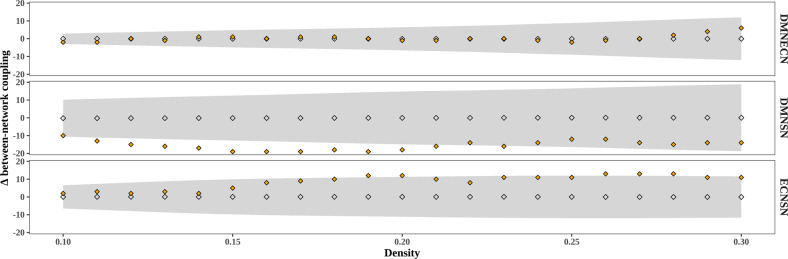
Difference in between-network maturational coupling between preterm-born infants exposed to high versus low stress (median split) across density levels. Positive values show high stress >low stress and negative values show low stress >high stress. Gray diamonds depict the permutation distribution of average maturational coupling, gray bands depict 95% confidence interval, and orange diamonds depict the group-level difference score.

**Fig. 3:**
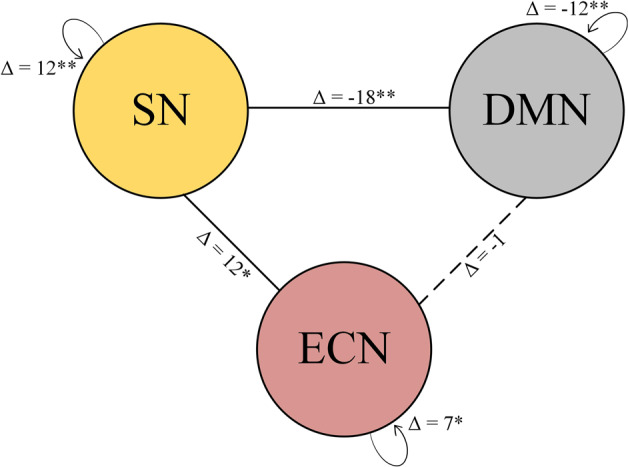
Graphical representation of network-level differences in maturational coupling. Delta constitutes the difference score in the number of covariance connections between high-stress-exposed infants and low-stress-exposed infants. Positive values indicate high stress >low stress. DMN default mode network, SN salience network, ECN executive control network. *P < 0.05, **P < 0.01.

We next examined between-network coupling. Our analysis did not show significant differences in DMN-ECN maturational coupling between the low and high-stress groups (∆ = −1, *p-FDR* = 0.896). However, infants exposed to high stress exhibited decreased between-network covariance between the SN and DMN (∆ = −18, *p-FDR* < 0.01), and between the SN and ECN (∆ = 7, *p-FDR* < 0.05). These findings suggest that “third-trimester” changes in MCNs are dependent on stress exposure, with high-stress exposure leading to developmental fluctuations in a network-specific manner (see Figs. 2 and 3). The between-network findings for DMN-SN were replicated across several density levels, but not all, and grouping criteria while the SN-ECN findings were less consistent (see Supplementary Figs. 1 and 2).

An important confounder is the administration of postnatal corticosteroids (for lung maturation). To ensure that the stress effects could not be attributed to non-stress aspects of the intensive-care environment, we repeated the analyses excluding infants who received postnatal corticosteroids (i.e., hydrocortisone). As shown in Supplementary Figs. 4 and 5, the higher coupling within the SN (∆ = 6, *p-FDR* < 0.05), the lower coupling within the DMN (∆ = −11, *p-FDR* < 0.01), and the decoupling between the DMN-SN (∆ = −17, *p-FDR* < 0.01) following high stress were replicated.

**Fig. 4:**
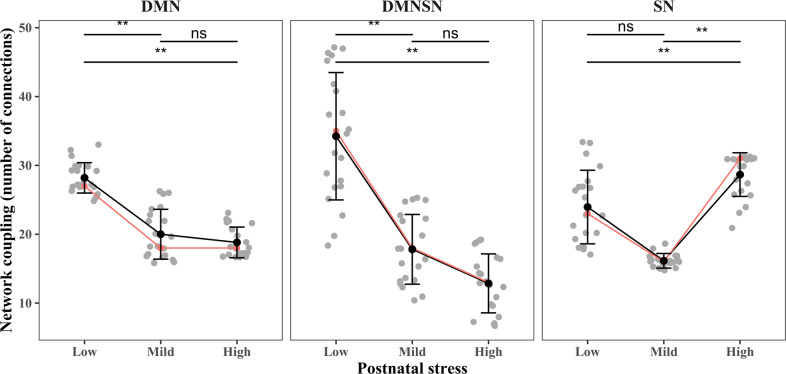
Differences in structural coupling for low (<33.3%), mild (33.3–66.6%), and high-stress-exposed infants (>66.6%). Observations are the number of connections using different density thresholds with mean (black dots) and standard deviation (error bars). The orange line depicts structural coupling at a network sparsity of 20%. DMN default mode network, SN salience network. **P < 0.01, ns non-significant.

We also explored the potential dose-dependent effects of early-life stress by categorizing infants into low, mild, and high-stress exposure. As shown in Fig. 4, a similar pattern was observed with a lower coupling within DMN and between DMN-SN, and a higher coupling within SN in infants exposed to more stress. Importantly, the decoupling within the DMN and between the DMN-SN showed the steepest decline from low to mild stress exposure (DMN: ∆ = −9, *p-FDR* < 0.01, DMN-SN: ∆ = −17, *p-FDR* < 0.01), whereas no changes were observed from mild to high-stress exposure. The increased coupling of the SN is only observed in infants exposed to the highest level of stress (low-high-stress difference: ∆ = 8, *p-FDR* < 0.01).

### The effects of early-life stress on region-level maturational covariance

We examined specific contributions of brain regions in the networks sensitive to stress-induced alterations. Significantly greater (FDR-corrected, *q* = 0.05) maturational coupling was observed in high-stress compared to low-stress infants within several homologous regions of the SN, namely between the right insula and bilateral amygdala (left: *Z* = 4.32, *p-FDR* < 0.001, right: *Z* = 3.72, *p-FDR* < 0.001), between the right subthalamic nucleus and bilateral insula (left: *Z* = 3.95, *p-FDR* < 0.001; right: *Z* = 4.32, *p-FDR* < 0.001), between interhemispheric amygdala (*Z* = 15.67, *p-FDR* < 0.001), and between the right amygdala and bilateral subthalamic nucleus (left: *Z* = 27.04, *p-FDR* < 0.001; right: *Z* = 18.04, *p-FDR* < 0.001).

Following high stress, maturational coupling was weaker for the DMN regions, specifically between the bilateral hippocampus (*Z* = −2.03, *p-FDR* < 0.05), between the bilateral anterior fusiform (*Z* = −12.09, *p-FDR* < 0.001), between the right posterior fusiform and bilateral anterior fusiform (left: *Z* = −11.68, *p-FDR* < 0.001; right: *Z* = −15.82, *p-FDR* < 0.001), and between the right posterior parahippocampal gyrus and bilateral anterior fusiform (left: *Z* = −12.41; right: *Z* = −15.23, *p-FDR* < 0.001), among others.

**Fig. 5:**
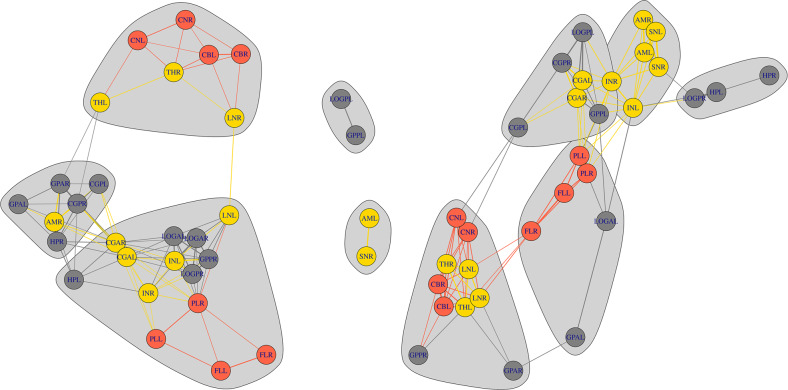
A schematic representation of the alterations in within- and between-network maturational covariance of preterm-born infants exposed to low (left) and high (right) stress. Gray; default mode network, yellow; salience network, red; executive control network. HPL hippocampus left, HPR hippocampus right, AML amygdala left, AMR amygdala right, GPAL parahippocampal gyrus anterior left, GPAR parahippocampal gyrus anterior right, LOGAL anterior fusiformis left, LOGAR anterior fusiform right, CBL cerebellum left, CBR cerebellum right, INR insula right, INL insula left, GPPR parahippocampal gyrus posterior right, GPPL parahippocampal gyrus posterior left, CGAR cingulate cortex anterior right, CGAL cingulate cortex anterior left, CGPR cingulate gyrus poster right, CGPL cingulate gyrus posterior left, CNR caudate nucleus right, CNL caudate nucleus left, THR thalamus right, THL thalamus left, SNR subthalamic nucleus right, SNL subthalamic nucleus left, LNR lentiform nucleus right, LNL lentiform nucleus left, FLR frontal lobe right, FLL frontal lobe left, PLL parietal lobe left, PLR parietal lobe right, LOGPL posterior fusiform gyrus left, LOGPR, posterior fusiform gyrus right. Network sparsity at 20%.

Significant lower maturational coupling was observed in infants exposed to high stress between several DMN-SN regions. Analyses revealed a lower coupling between the left insula and right posterior parahippocampal gyrus (*Z* = −16.04, *p-FDR* < 0.001) and between the left insula and right posterior fusiform gyrus (*Z* = −17.91, *p-FDR* < 0.001). In addition, high-stress-exposed infants showed decreased synchronized development between the bilateral anterior cingulate cortex and the right posterior parahippocampal gyrus (left: *Z* = −3.75, *p-FDR* < 0.001; right: *Z* = −3.29, *p-FDR* < 0.001). A schematic representation of the altered within- and between-network maturational coupling is shown in Fig. 5. For a complete overview of region-level alterations, see Supplementary Table 1.

## Discussion

The current study characterized maturational covariance patterns of three large-scale brain networks in a sample of extremely preterm-born infants, scanned at 30 and 40 weeks of gestation, exposed to ex utero “third-trimester” stress. Infants exposed to high stress showed higher maturational covariance for the SN and a reduced covariance for the DMN. In addition, the high-stress-exposed infants showed lower maturational covariance between the SN and DMN. Follow-up analyses showed a more nuanced pattern such that a decoupling within the DMN and between the DMN-SN was already observed in infants exposed to mild stress, whereas an increased coupling of the SN was only observed in infants exposed to the highest level of stress (top 66.6%). The current findings indicate that “third-trimester” stress exposure leads to a reprioritization of developmental trajectories by altering developmental covariance patterns in a network-specific manner.

The association between early-life stress exposure and alterations in brain development has been supported by prior studies [21, 50]. The current study investigates the notion of a developmental trade-off, such that alterations in the brain’s salience processing network may delay the development of other brain networks. Understanding whether this trade-off exists provides a potential pathway to maladaptive behavior following early-life stress and prematurity.

Several studies suggested that maturational covariance patterns reflect regions that functionally coactivate [22, 51], with functional networks guiding the maturation of covariance patterns [23]. Consistent with previous studies on the functional development following early stressful experiences (e.g., trauma, insensitive parenting, prematurity [15, 16, 50, 52–54]), with a higher coupling of the amygdala and insula, we found that the maturational coupling of the SN, a network that subserves the processing of emotions, is significantly influenced by “third-trimester” stress exposure. High-stress-exposed infants showed a higher coupling between the amygdala, insula, and subthalamic nucleus. On the contrary, infants exposed to high stress showed a lower covariance within the DMN, specifically a lower coupling between the hippocampus, parahippocampal gyrus, and fusiform.

Animal studies on the potential mechanisms underlying the effects of chronic early-life stress on brain development report profound and region-specific developmental decline of cell proliferation and neurogenesis, as well as an increased cell death (e.g., doublecortin [DBC], Ki-67 [55–57]). More recent studies reported that chronic stress also promotes an earlier rise in myelin basic protein expression, increases synaptic maturation (N-methyl-d-aspartate [NMDA] receptor subunits), and accelerates the emergence of interneurons (parvalbumin [PV] cells) [58–60]. Interestingly, PV expression has been implicated in the opening and closure of critical periods, also called the “plasticity switch” [18, 61, 62]. This means that in response to external factors, PV cells might mature faster and lead to a precocious onset of a critical period, shifting neural circuits from an immature to a plastic state. During such a critical period, the brain is open to circuit rewiring based on input from the environment. Hence, early-life stress exposure possibly promotes activity-dependent modification in cellular events, and in turn, regulates the rewiring of the brain’s salience processing networks.

Structural and functional aspects of the amygdala, subthalamic nucleus, and insula represent salient features, including perceptual vigilance, novelty, aversion, and arousal [63–65]. The higher maturational coupling within SN-specific nodes might reflect heightened arousal, supporting the anticipation and processing of salient stimuli. Indeed, some studies showed an association between heightened insula-amygdala-subthalamic nucleus coupling and higher anxiety-related disorders [66, 67]. Although the behavioral literature in preterm-born individuals is limited, a few studies found evidence of more extensive processing of salient stimuli in preterm-born infants [68, 69]. Speculative, these studies, combined with our present findings, suggest that higher SN maturational coupling following “third-trimester” stress might facilitate a heightened vigilance and arousal, which could be considered an adaptive response considering the environmental salient experiences during NICU admission.

This study also presents lower maturational covariance within the DMN following high stress, specifically between the bilateral hippocampus, parahippocampal gyrus, and fusiform. These findings are in line with prior studies reporting reduced structural and functional coherences of the DMN in preterm-born infants [70–73], children [74], and adults [75]. Studies on early-life trauma have repeatedly established a link between reduced DMN coupling and future stress-related psychopathologies, including PTSD, anxiety, and depression [76–78]. Reduced DMN coherence following early-life stress might reflect an inability to allocate resources properly between internal thoughts and external stimuli and might be related to impaired internal monitoring and less optimal emotional regulation capacity [79, 80]. Findings of increased difficulty in regulating emotion and arousal and the flexible allocation of attention in preterm-born individuals support this interpretation [81].

The SN has a modulatory role in switching between the DMN-ECN, highlighting the dynamic interaction between the three networks [82]. We found alterations in the between-network maturational coupling of the DMN-SN following early-life stress exposure, suggesting a disruption of the neural equilibrium. Our results showed lower maturational covariance between the SN and DMN following high stress, including a lower coupling between the insula, parahippocampal gyrus, and fusiform gyrus, amongst others. These findings replicate prior functional studies reporting a hypoconnectivity between the insula/amygdala and DMN at term equivalent age [83], adolescence [54], and adulthood [75, 84]. As suggested by the authors, lower coupling between the DMN-SN could indicate an overactive inhibitory function of the DMN in modulating the SN, significantly affecting one’s emotional processing [84]. This interpretation receives further support from findings that show that preterm infants exhibit decreased sustained attention (distractibility), increased avoidance behavior, and fear later in life [68, 85–87]. Importantly, as the proposed association between the observed alteration in SN and DMN coupling and the functional outcome remains purely speculative, more research is needed to disentangle the behavioral consequences and its (mal)adaptive function.

It remains elusive whether SN-related changes represent an accelerated maturation prioritizing adult-like functioning (i.e., stress-acceleration hypothesis [21, 50]) or a compensatory strategy in a suboptimal efficient network. The vulnerability of the SN and DMN might be a byproduct of the malleability required to cope with adverse stimuli early in life. Given this, the developmental trade-off might be interpreted as beneficial in a population of extremely preterm-born infants as the NICU environment often prohibits the caregiver from providing external regulation. The behavioral literature indeed suggests that early-life stress might enhance an adaptive defensive phenotype in response to a threat, as indicated by the increased conscious awareness of negative stimuli. More specifically, preterm infants can identify stimuli as salient and show heightened affective negativity [88], less social engagement, and increased gaze aversion compared to full-term controls [89]. Though these defensive behaviors might be adaptive in the short term, e.g., avoiding physical harm, alterations may serve an increased risk for future functional impairments.

Life-history theory argues that exposure to threat and violence, a harsh environment, favors an accelerated maturation, as opposed to a deprived environment in which resources are conserved [90]. A natural implication of this theory is that threat and neglect might shape health and development in disparate and possibly even opposing ways [91–93]. Despite striking similarities in structural/functional coupling for populations exposed to a variety of stressors [15, 16, 50, 52–54], with parallels in (maternal) childhood trauma, maternal stress, insensitive parenting, and also prematurity, the extent to which the network-specific alterations may be specific to stress characterized by threat is less clear. Empirical evidence is scarce, and studies that did investigate the diverging effects of threat and neglect are too diverse to compare, both conceptually and methodologically [94–97]. Consequently, more research is needed to investigate whether and how the effects of different types of stressors are outcome-specific and increase our understanding of the functional consequences of altered maturational coupling following early-life stress and prematurity.

The ECN seemed to be relatively unaffected by the degree of “third-trimester” stress exposure. One explanation could be considerable variability in spatiotemporal development, with profound differences in developmental timing of maturation of these brain regions. In a recent review [2], we found preliminary evidence for a developmental sequence starting from the DMN to the SN, and finally the ECN. Nodes implicated in the DMN and SN appear to be maturing faster, namely during the beginning of the first trimester, contrarily to the relatively delayed maturation of cortical nodes implicated in the ECN. In other words, early-life stress might impact ECN in a delayed fashion, such that “third-trimester” imaging is unable to capture the alterations in the developmental trajectory. Alternatively, there might be differing sensitivity of the ECN to stress signals through the differing regional expression of glucocorticoid (GR) and mineralocorticoid receptors (MR). Studies for instance reported an age-related increase in MR and GR expression for the frontal lobe, with lower expression being observed in infancy than in childhood and adolescence [98, 99].

There are a number of limitations that need to be taken into consideration when interpreting our findings. First, due to the group-averaged MCNs, the influence of inter-subject variability could not be reliably assessed. Future longitudinal studies are needed to identify individual- and population-based trajectories precisely. Also, the current statistical approach of group-averaged MCNs only allowed for the categorization of stress. The dichotomization of continuous variables leads to reduced power, lost information, and an increased probability of false negatives. The alterations in within- and between-network maturational coupling following early-life stress were substantially similar across a range of density levels and remained significant using a different cut-off point for the degree of stress exposure for the within-network findings, and to a lesser degree for the between-network findings (i.e., zero-mean, Supplementary Figs. 2 and 3). Lastly, the preterm-born population is increasingly susceptible to (chronic) disease, and the degree of illness and stress exposure is often tightly linked. Although we adjusted for clinical confounders of prematurity, it remains a challenge to disentangle the effects of illness and stress, and uncontrolled confounders (including sleep, parent-infant relationship, and nutrition [100–102]) might distort our interpretation in a variety of ways. To ensure that the network-specific changes are not due to non-stress-related aspects of the medical environment, we repeated the analyses and removed infants who received postnatal corticosteroids. We found similar network-specific alterations in infants exposed to high stress (see Supplementary Figs. 4 and 5). However, the included confounders related to clinical conditions during the NICU stay are not exhaustive.

Despite these limitations, our study comprehensively identifies within- and between-network maturational covariance patterns following “third-trimester” stress exposure in extremely preterm-born infants. Our findings indicate that early stress may lead to a reprioritization of developmental trajectories, as higher “third-trimester” stress leads to higher maturational coupling within regions of the SN, lower coupling within regions of the DMN, and a decoupling between the DMN-SN. This developmental trade-off may enhance the ability to cope with adverse stimuli early in life and simultaneously render individuals at a higher risk of developing later stress-related psychopathology.

## Supplementary information

Supplementary Information

Figure 1

Figure 2

Figure 3

Figure 4

Figure 5

Table 1
