# Correction: Early-life stress exposure and large-scale covariance brain networks in extremely preterm-born infants

**DOI:** 10.1038/s41398-022-02036-3

**Published:** 2022-07-08

**Authors:** Femke Lammertink, Martijn P. van den Heuvel, Erno J. Hermans, Jeroen Dudink, Maria L. Tataranno, Manon J. N. L. Benders, Christiaan H. Vinkers

**Affiliations:** 1 Department of Neonatology, University Medical Center Utrecht, Utrecht University, Utrecht, The Netherlands; 2 Department of Complex Trait Genetics, Center for Neurogenomics and Cognitive Research, Amsterdam Neuroscience, Vrije University Amsterdam, Amsterdam, The Netherlands; 3 Department of Child Psychiatry, Amsterdam Neuroscience, Amsterdam UMC, Amsterdam, The Netherlands; 4 Donders Institute for Brain, Cognition, and Behaviour, Radboud University, Nijmegen, The Netherlands; 5 Department of Cognitive Neuroscience, Radboud University Medical Center, Nijmegen, The Netherlands; 6 Department of Anatomy & Neurosciences, Amsterdam UMC (location Vrije University Amsterdam), Amsterdam, The Netherlands; 7 Department of Psychiatry, Amsterdam UMC (location Vrije University Amsterdam), Amsterdam, The Netherlands

**Keywords:** Neuroscience, Psychiatric disorders

Correction to: *Translational Psychiatry* 10.1038/s41398-022-02019-4, published online 18 June 2022

Supplementary figures and supplementary table were missing from this article.

Moreover, the title for Fig. 2 should have appeared as below.

Difference in between-network maturational coupling between preterm-born infants exposed to high versus low stress (median split) across density levels.

The original article has been corrected.

## Supplementary information

Figure S1

Figure S2

Figure S3

Figure S4

Figure S5

Table S1

