# Supplementary material for: Early-life stress exposure and large-scale covariance brain networks in extremely preterm-born infants
Source: Transl Psychiatry. 2022 Jun 18;12:256. doi: 10.1038/s41398-022-02019-4 (PMC9206645; doi:10.1038/s41398-022-02019-4)
Supplement: Supplementary file 1 — Supplementary Information [file 41398_2022_2019_MOESM1_ESM.docx]

**Supplementary Information**

Early-life stress exposure and large-scale covariance brain networks in extremely preterm born infants

Lammertink et al

*Supplementary Table S1.* Fisher’s r-to-z transformation of difference in maturational covariance between low and high-stress exposed infants. Higher values indicate an increased maturational coupling in high—stress-exposed infants compared to low-stress exposed infants. Lower triangle depicts the Z-values, upper triangle depicts significance level. Network sparsity at 20%.

*Supplementary Figure S5.* Subsample of infants who did not receive postnatal corticosteroids (*N*=126). Difference in between-network maturational coupling between preterm-born infants exposed to high versus low-stress across density levels. Positive values show high stress > low stress and negative values show low stress > high stress. Grey diamonds depict the permutation distribution of average maturational coupling, grey bands depict 95% confidence interval, and orange diamonds depict the group-level difference score.

*Supplementary Figure S4.* Subsample of infants who did not receive postnatal corticosteroids (*N*=126). Difference in within-network maturational coupling between preterm-born infants exposed to high versus low-stress across density levels. Positive values show high stress > low stress and negative values show low stress > high stress. Grey diamonds depict the permutation distribution of average maturational coupling, grey bands depict 95% confidence interval, and orange diamonds depict the group-level difference score.

*Supplementary Figure S3.* Difference in between-network maturational coupling between preterm-born infants exposed to high versus low-stress (zero-mean split) across density levels. Positive values show high stress > low stress and negative values show low stress > high stress. Grey diamonds depict the permutation distribution of average maturational coupling, grey bands depict 95% confidence interval, and orange diamonds depict the group-level difference score.

*Supplementary Figure S2.* Difference in within-network maturational coupling between preterm-born infants exposed to high versus low-stress (zero-mean split) across density levels. Positive values show high stress > low stress and negative values show low stress > high stress. Grey diamonds depict the permutation distribution of average maturational coupling, grey bands depict 95% confidence interval, and orange diamonds depict the group-level difference score.

*Supplementary Figure S1.* Distribution (median, range) of (A) duration of NICU admission and (B) total number of invasive procedures prior to the first scan (30 weeks of gestation).
