# Supplementary figures and images for: Early-life stress exposure and large-scale covariance brain networks in extremely preterm-born infants
Source: Transl Psychiatry. 2022 Jun 18;12:256. doi: 10.1038/s41398-022-02019-4 (PMC9206645; doi:10.1038/s41398-022-02019-4)

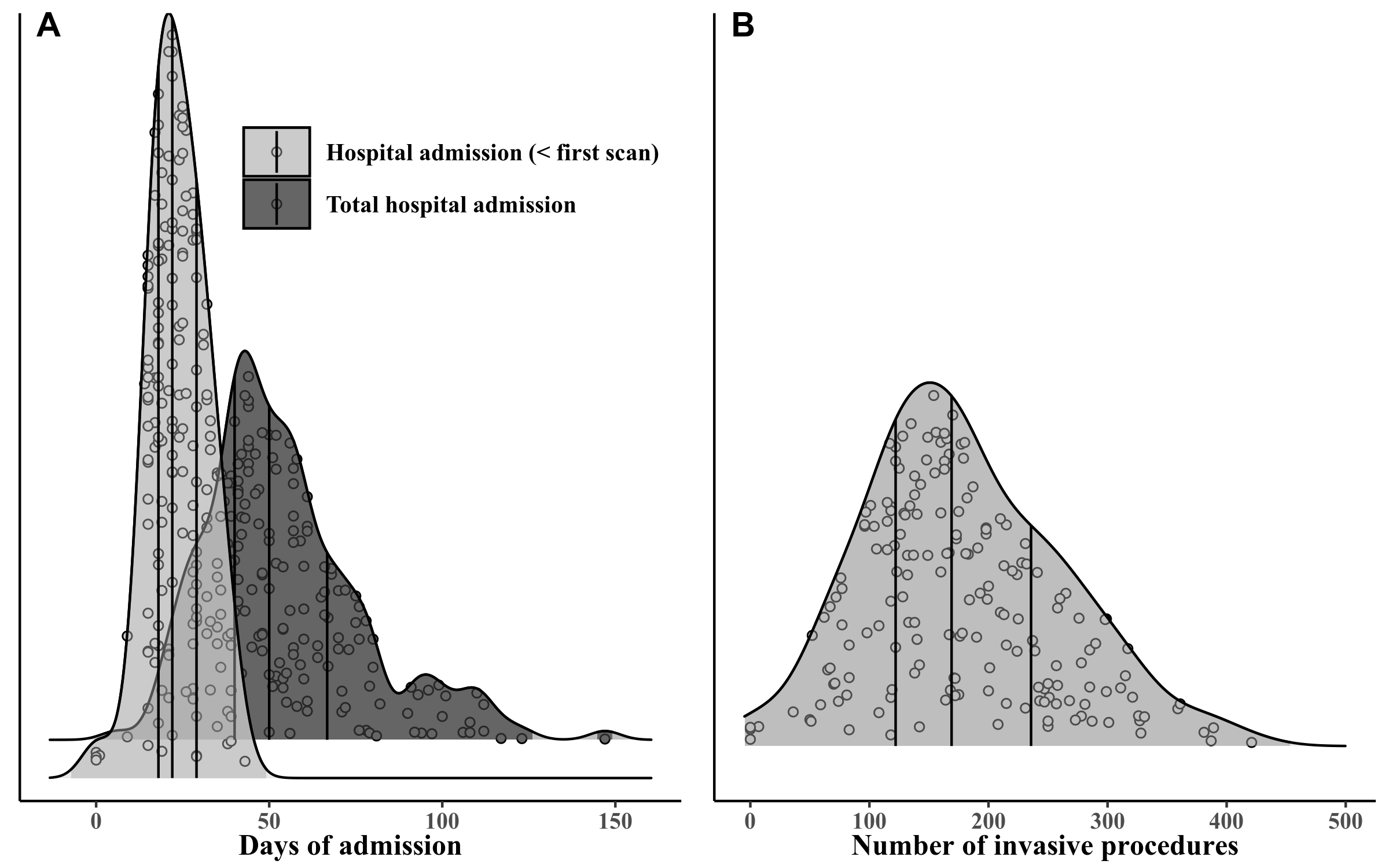

Supplement: Supplementary file 2 — Figure 1 [file 41398_2022_2019_MOESM2_ESM.tiff]

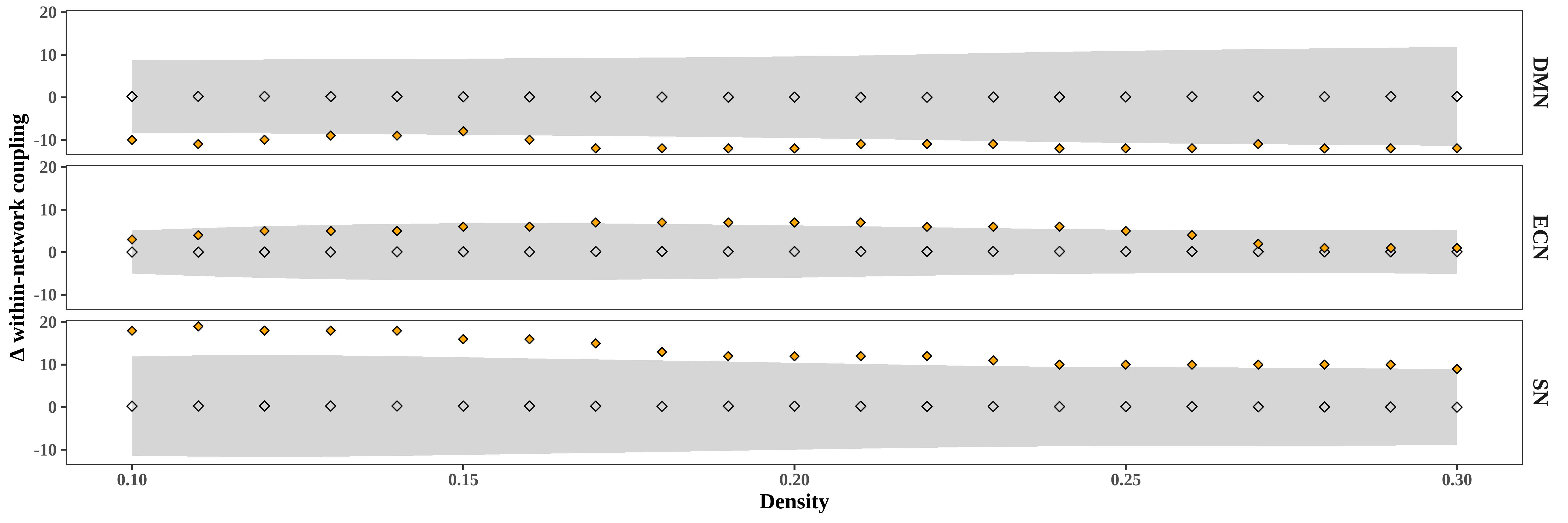

Supplement: Supplementary file 3 — Figure 2 [file 41398_2022_2019_MOESM3_ESM.tiff]

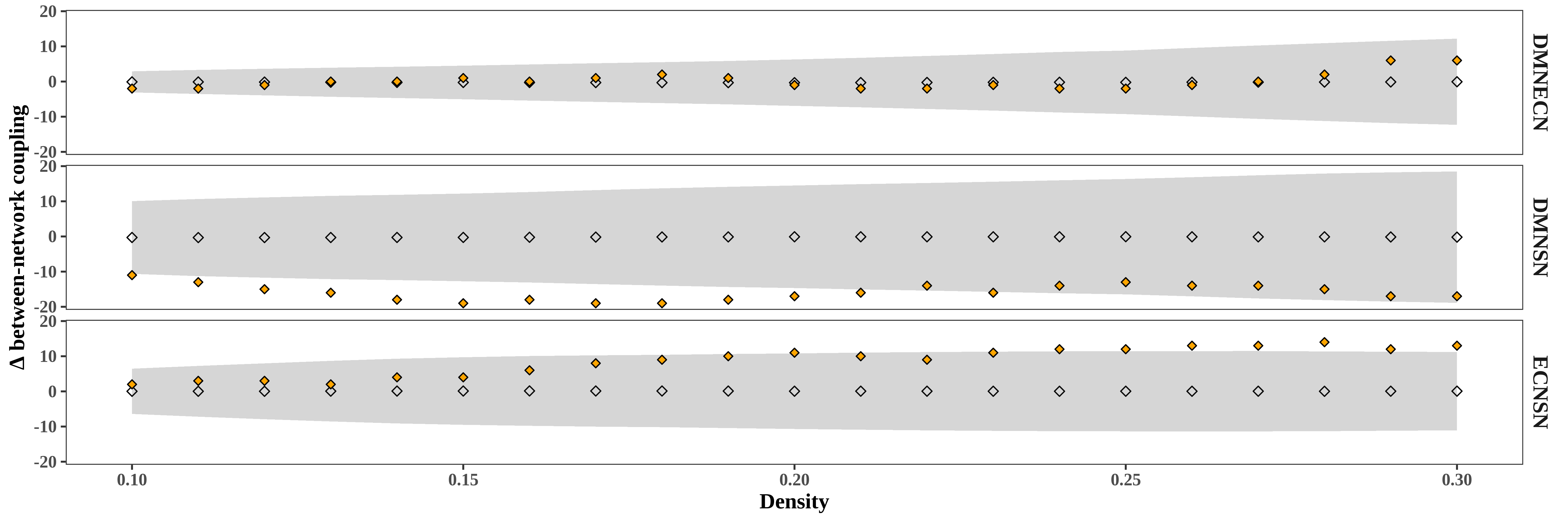

Supplement: Supplementary file 4 — Figure 3 [file 41398_2022_2019_MOESM4_ESM.tiff]

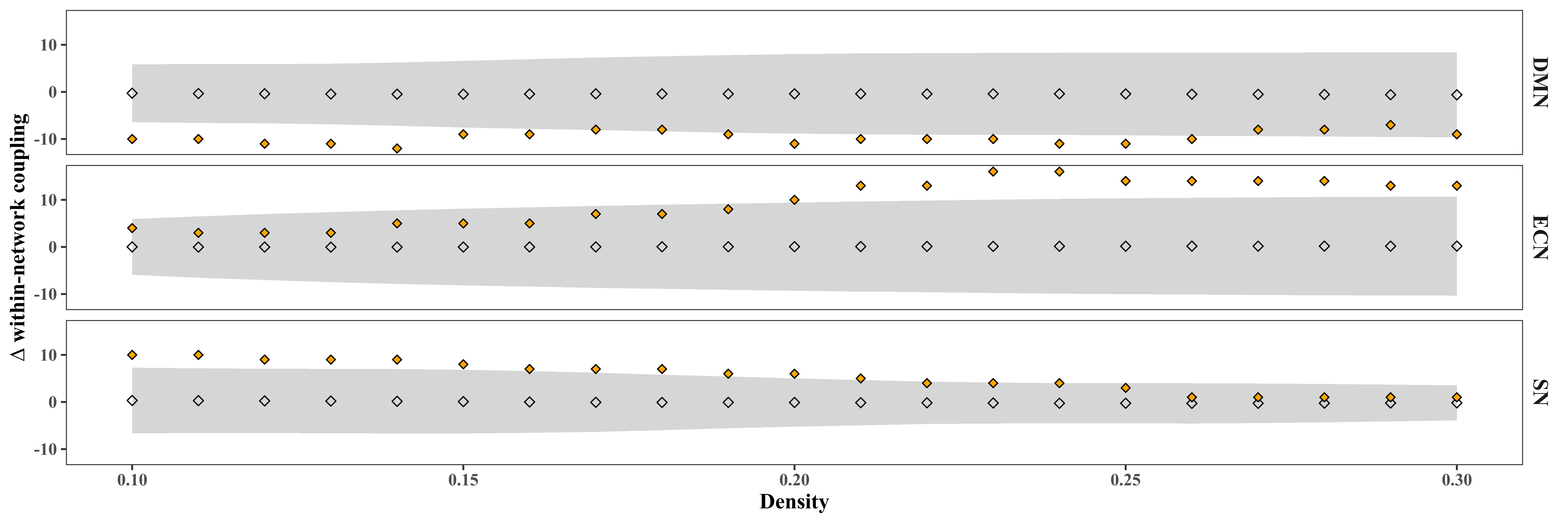

Supplement: Supplementary file 5 — Figure 4 [file 41398_2022_2019_MOESM5_ESM.tiff]

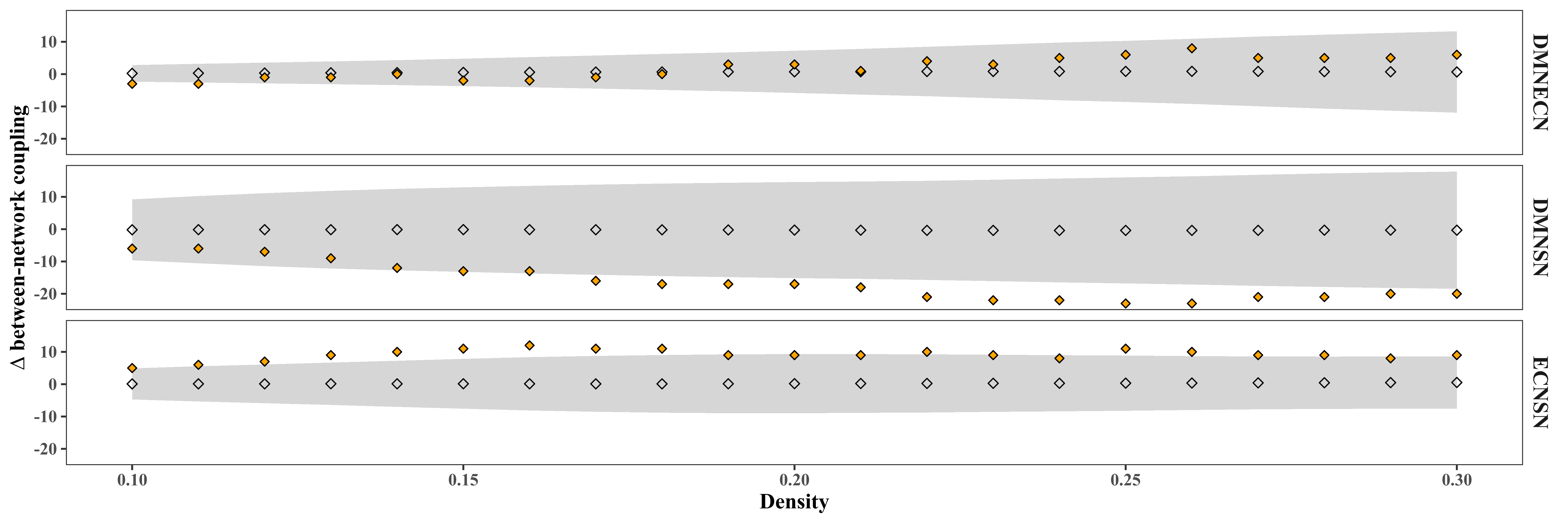

Supplement: Supplementary file 6 — Figure 5 [file 41398_2022_2019_MOESM6_ESM.tiff]
